# The Self-Figure Drawing Emotional Abuse Index: A Preliminary Convergent Validity Study with Teacher-Reported Neglect and Psychological Adjustment in Italian Children

**DOI:** 10.3390/children13101438

**Published:** 2026-10-20

**Authors:** Matteo Angelo Fabris, Meghna Girish

**Affiliations:** 1 Department of Psychology, University of Turin, 10124 Turin, Italy; 2 School of Creative Arts Therapies, University of Haifa, Haifa 3498838, Israel

**Keywords:** emotional abuse, neglect, self-figure drawing, convergent validity, psychological adjustment, school-based screening

## Abstract

**Highlights:**

**What are the main findings?**

- In a community sample of 392 Italian children aged 8–10, 14.3% showed Self-Figure Drawing (SFD) indicators of emotional abuse, and the SFD emotional abuse index was significantly, albeit modestly, associated with teacher-reported neglect and overall psychological difficulties.
- These results provide the first preliminary evidence of the convergent validity of the SFD emotional abuse index outside its original cultural context, using independent teacher-report measures in an Italian sample.

**What is the implication of the main finding?**

- The SFD may serve as a complementary, non-verbal screening tool to help school professionals flag children potentially at risk of emotional abuse, alongside teacher observation and other assessment methods.
- Given the modest effect sizes and single-informant design, further validation with multiple informants and clinically confirmed samples is needed before the SFD can be considered for applied screening use.

**Abstract:**

**Background/Objectives:** Emotional abuse and neglect remain comparatively understudied forms of child maltreatment, and non-verbal screening tools such as the Self-Figure Drawing (SFD) have rarely been examined outside their original cultural contexts. This study provided a preliminary test of the convergent validity of the SFD emotional abuse (EA) index with independent teacher-report indicators of neglect and psychological adjustment in an Italian community sample. **Methods:** Participants were 392 Italian children aged 8–10 years. Children completed a Self-Figure Drawing, which was coded for emotional abuse indicators by two trained independent raters blind to teacher reports; teachers completed a neglect scale and the Strengths and Difficulties Questionnaire (SDQ), whose Total Difficulties score was used as an indicator of global psychological difficulties (higher scores indicated poorer adjustment). **Results:** A total of 14.3% of children met SFD criteria for emotional abuse. Correlational analyses showed that the EA index was significantly, albeit modestly, associated with both teacher-reported neglect and the SDQ Total Difficulties score, indicating that children with more pronounced emotional abuse indicators on their drawings were also rated by teachers as more neglected and as showing greater overall psychological difficulty. **Conclusions:** These findings offer preliminary evidence that the SFD emotional abuse index converges with independent, informant-based indicators of maltreatment and psychological difficulties in an Italian community sample. Implications for the use of projective drawing measures in school-based screening for emotional abuse, and directions for further validation of the SFD in Italian samples, are discussed.

## 1. Introduction

Child abuse and maltreatment remain pervasive social issues worldwide, including in Italy [1,2,3]. Among the various forms of child abuse and maltreatment, international research indicates that emotional abuse is one of the most widespread forms of victimisation [4,5]. In Europe, an estimated 55 million individuals under the age of 18 have been exposed to emotional abuse within the family, compared with 44 million exposed to physical abuse and 18 million exposed to sexual abuse [4]. Emotional abuse is strongly associated with the risk of physical and emotional neglect [6], to the extent that the literature has proposed the construct of emotional maltreatment to capture the combination of the two [7,8]. Whereas emotional abuse includes verbal aggression, belittlement, and rejection, emotional neglect is characterised by psychological or emotional distancing and a failure to meet the child’s emotional needs [9,10]. Looking specifically at Italy, nearly half of the children under the care of social services for family maltreatment are reported to experience physical and psychological neglect, a proportion considerably higher than the European average (around 35%) [11,12]. This finding illustrates how emotional abuse and its associated experiences of neglect constitute an ongoing emergency within the Italian context. However, despite growing research interest in emotional abuse among children, this form of childhood victimisation remains markedly understudied compared with other forms of violence against minors, such as physical and sexual abuse [6]. This is particularly concerning given that emotional abuse is associated with increased psychological distress in children [13,14] and can affect developmental outcomes well into adolescence and adulthood [15], including depressive and anxiety symptoms, eating and body-image disorders, personality disorders, and suicidal ideation [14,15,16]. Several studies have found that individuals from clinical populations report higher levels of emotional abuse and emotional neglect than those from non-clinical populations [15]. Moreover, compared with more overt forms of abuse such as physical and sexual abuse, emotional abuse is not only associated with a wider range of negative developmental outcomes and poorer quality of life [17], but also emerges as the strongest independent predictor of psychiatric symptoms in studies that simultaneously control for multiple forms of child maltreatment [18]. Emotional abuse also predicts the highest rate of subsequent revictimisation, with a 40% increase in the average number of later traumatic events, suggesting that it undermines the emotion regulation abilities and interpersonal skills needed to recognise and avoid threats [19].

Emotional abuse should also be understood within the broader context in which it occurs. Ecological and transactional models of child maltreatment conceptualise abuse as the outcome of interacting risk and protective factors operating at the individual, family, community, and societal levels [20,21]. Among these, poverty and socioeconomic disadvantage are among the most consistently documented contributors: economic hardship exposes parents to chronic stress and degrading living conditions, which, according to the family stress model, can undermine parents’ emotional availability and increase harsh, hostile, or rejecting parenting [22]; consistently, a substantial body of evidence links family poverty to a higher risk of child abuse and neglect [23]. Factors at different levels may also interact. For instance, schools with limited resources may be less able to support families under strain or to provide children with compensatory relationships and opportunities. From a critical realist perspective, such factors can be understood as contributing and counteracting mechanisms whose interplay determines whether a given risk results in an observable outcome [24]: a supportive school environment and a close teacher–child relationship [25,26] may, for example, partly compensate for a family’s difficulty in providing emotional support, thereby attenuating—or making less visible—the consequences of emotional abuse. Although these structural determinants were not the focus of the present study, they provide an important framework for interpreting school-based indicators of emotional abuse.

Given this background, it is increasingly important to devote greater attention to the prevention and identification of emotional abuse in childhood, and in this respect, schools represent an important observational setting [27]. School is the extra-familial context in which children spend the greatest amount of time and, given the considerable number of hours they spend there, it represents a setting in which teachers can observe the child, receive disclosures and accounts of family experiences, and notice concerning behavioural changes or worries [28,29]. It should also be considered that teachers can function as attachment-like figures for children [25], meaning that students who experience closeness and trust with their teachers may turn to them for help regarding forms of victimisation, or may confide their feelings and family-related difficulties to them [26]. However, several characteristics appear to make emotional abuse a particularly under-detectable form of victimisation, even within educational settings. In general, emotional abuse does not occupy the same space as other forms of maltreatment in public discourse, and people tend to consider it less harmful to children than physical or sexual abuse [30,31]. Furthermore, the literature describes emotional abuse as an “invisible” form of maltreatment, in the sense that it produces intrapsychic wounds with few externally observable markers [32], and that it can be difficult for professionals working with children to draw a clear line between emotional abuse and poor parenting [33]. These aspects are important to consider, as they may affect teachers’ and educators’ ability to correctly identify situations of emotional abuse and, crucially, to fulfil their obligation to report cases of victimisation to the relevant authorities. Some evidence supports this view. For example, in an experimental vignette study, the accuracy of emotional abuse detection was 86%, but the corresponding reporting rate dropped to 70%, indicating that a proportion of recognised cases are not subsequently reported [34]. Teachers appear to feel more confident addressing more visible forms of maltreatment, particularly physical maltreatment [35], and tend to under-report cases of emotional abuse, especially those perceived as less severe [36]. From the child’s perspective, moreover, victims of emotional abuse often find it difficult to recognise their own experiences as abusive, instead perceiving them as normal dynamics typical of their family environment, which may make young victims more reluctant to disclose their experiences of victimisation [37,38]. Taken together, detecting cases of childhood emotional abuse therefore remains a considerable challenge, underscoring the need to develop screening tools capable of flagging the need for further assessment or closer attention to a child’s life experience.

One of the observational tools most widely used in clinical and educational settings to access children’s inner world is children’s drawing [39,40]. Within educational and psychological research, children’s drawing has been extensively used as a tool capable of capturing children’s representations and knowledge of important or emotionally salient topics, allowing educators and researchers to understand how children interpret phenomena and what they know about them [41,42]. For children, producing a drawing is typically an enjoyable activity and may therefore be more motivating and engaging than completing a self-report questionnaire [39,43]. However, children’s drawing is not only a valuable research tool but has traditionally also been used as an instrument for the psychological assessment of children in clinical and educational contexts [39,40]. From this perspective, children’s drawing is regarded as a graphic-projective tool capable of accessing the child’s inner world, capturing representations linked to mental states, conflicts, and feelings [40,44]. Furthermore, children’s drawing allows children to express themselves through a non-verbal channel, enabling them to overcome language barriers and also enabling comparisons between groups with different languages and abilities [45]. Given these potential strengths, children’s drawing may therefore serve as an unobtrusive tool for detecting situations of potential victimisation risk, such as emotional abuse.

In this respect, one instrument that has proven particularly sensitive in detecting children potentially at risk of emotional abuse is the Self-Figure Drawing test (SFD [46]), developed to provide a non-verbal screening tool able to overcome children’s reluctance to disclose adverse experiences. Furthermore, several studies conducted across different cultural contexts have shown the usefulness of the SFD in identifying children and adolescents experiencing elevated psychological distress [47,48]. Clinical experts and art therapy researchers argue that drawings enable the expression of hidden or repressed thoughts and feelings in a relatively fast and straightforward way [49]. In this sense, drawings can shed light on children’s internal and external worlds and on their perception of their families and parents, communicating feelings and ideas about their environment [49,50]. Moreover, clinical and empirical evidence indicates that children’s drawings, particularly those produced during a time of crisis, can overcome children’s language limitations, which makes the SFD a particularly interesting graphic tool for the assessment of children in school settings who may potentially have been exposed to maltreatment and abuse [48,51]. Because emotional abuse can constitute a painful and hard-to-verbalise experience for a child, the SFD provides children with an alternative means of depicting emotions, relationships, and experiences that may be inaccessible through words alone [51]. The SFD was developed by Lev-Wiesel and colleagues [46,52] on the basis of the well-known Draw-a-Person Test (DAP) by Machover [53]. Unlike the DAP, in which individuals are asked to draw a person, the SFD asks respondents to draw themselves [46]. Previous studies have identified several graphic indicators that appear to be frequently associated with experiences of emotional abuse in children and adolescents, specifically shading of the face, gross asymmetry of limbs, slanted figures, smaller bodies, tiny heads, omission of the eyes, and omission of the hands [38,54]. In this respect, the SFD therefore appears to be a usable tool within school settings for identifying children potentially at risk of emotional abuse. Naturally, the broader scientific status of projective drawing tests calls for caution in the use of instruments such as the SFD [55]. A child’s drawing should in no way be regarded as providing proof of abuse; rather, the potential of the test should be understood as that of a complementary screening tool within a broader observational protocol. The SFD has been applied across multiple cultural contexts, including Israel, Thailand, India, Slovakia, Argentina, and Spain, yielding results that support its cross-cultural applicability while also revealing significant variation [47,51]. For this reason, it is important to extend this line of research to new and previously unexplored cultural contexts, such as Italy. To date, the SFD has not, to our knowledge, been examined in an Italian general-population sample of school-aged children, nor has it been systematically related to independent, non-drawing-based indicators of maltreatment such as teacher-reported neglect or standardised measures of psychological adjustment. This represents a meaningful gap: establishing whether a drawing-based screening tool converges with independent informant reports is a necessary step before the tool can be considered for applied, school-based screening in a new cultural context. In the present study, teacher-reported neglect was selected as a convergent measure for emotional abuse not only because childhood emotional abuse and neglect are strongly associated in the literature, but also, and perhaps more importantly, because while emotional abuse produces an internal wound that is difficult to observe externally, neglect is more readily observable by teachers and may therefore represent an indicator that is more proximal to the risk of emotional abuse.

### Aim of the Study

The present study focuses specifically on the SFD emotional abuse (EA) index and examines its association with two informant-based measures collected independently of the drawing coding: teachers’ reports of neglect and teachers’ reports of children’s psychological difficulties. Rather than treating the SFD as a stand-alone prevalence-estimation tool, the present study aims to test the degree to which the SFD emotional abuse index converges with these two independent measures, providing an initial test of the instrument’s convergent validity in an Italian community sample of primary school children. The decision to focus on this sample was driven not only by the researchers’ specific interest but also by evidence that this age group is the most critical in terms of the potential impact of emotional abuse on children’s psychological adjustment and their development into adulthood [56].

Based on the theoretical overlap between emotional abuse and neglect, and on prior evidence that informant-based measures are more likely to capture chronic, environmentally embedded difficulties than acute or covert forms of harm, it was hypothesised that (H1) the SFD emotional abuse index would show a positive, albeit modest, association with teacher-reported neglect, and (H2) that it would similarly show a positive association with teacher-reported psychological difficulties (i.e., higher SDQ Total Difficulties scores), reflecting greater overall psychological maladjustment among children with more pronounced emotional abuse indicators.

## 2. Methods

### 2.1. Participants and Procedure

To conduct this study, several schools in northern Italy were contacted. The principals were contacted via email, and appointments were scheduled to explain the purpose and methodology of the research. After obtaining each principal’s approval, an informed consent form was sent to families for their children’s participation in the study. Only students who had received parental authorisation, spoke Italian, and possessed adequate cognitive and motor skills to participate in the study were recruited. No compensation was offered for participation. In addition to parental consent, written assent was obtained from each child using an age-appropriate form. The drawing task was administered in the classroom by a member of the research team, who explained to the children, in simple language, the purpose of the activity, that participation was voluntary, and that they could decline to take part or stop at any time without any consequences. Teachers were asked to complete questionnaires on their students’ psychological distress and risk of neglect. The children, meanwhile, were asked to draw a picture of themselves. The drawings were subsequently coded by two independent raters (see the Self-Figure Drawing section below); teachers did not code or score the drawings. To complete the drawing, the children were provided with a blank sheet of paper, a pencil, and coloured crayons. No time limit was set, considering the children’s ages. The study was approved by the Ethics Committee of the University of Turin and was conducted in accordance with the principles of the Declaration of Helsinki and the guidelines of the Italian Psychological Association (AIP). Given the sensitive nature of the study, a procedure for handling concerning information was established with the participating schools before data collection: should a child make a direct disclosure of maltreatment, or should other concerning information emerge, the research team would promptly inform the school principal and the school psychologist, who would activate the reporting procedures required by Italian law, under which teachers and school staff are obliged to report suspected child maltreatment to the competent authorities. No direct disclosures or concerning situations requiring the activation of this procedure occurred during the study. A total of 404 children aged 8–10 years participated in the study. After cleaning the dataset for missing values, the final sample size was 392 (*M* = 8.93 years; *SD* = 0.84). Children were recruited from 13 primary schools (30 classrooms). The questionnaires were completed by 30 teachers (one per classroom), each of whom rated the participating pupils in their own class; the number of children rated per teacher ranged from 2 to 27 (*M* = 13.07, *SD* = 5.68).

### 2.2. Measures

#### 2.2.1. Sociodemographic Data

The demographic sheet collected information on the child’s age, gender, whether the child had special educational needs, learning disorders, or physical or cognitive disabilities, as well as the parents’ marital status.

#### 2.2.2. Neglect

An 8-item scale [57] was used to assess the teacher’s perceived level of neglect of the child in the school context. The scale asked the teachers how they perceived the children on aspects such as being adequately dressed for the weather conditions, the child’s performance in class, the child’s food, hygiene and health. Each item is rated on a 5-point scale ranging from never (0) to always (4). Cronbach’s alpha for the scale in the current study was 0.74.

#### 2.2.3. Strengths and Difficulties Questionnaire (SDQ)

The SDQ [58,59] is a 25-item questionnaire that assesses children’s psychological adjustment through five subscales of five items each: emotional symptoms, conduct problems, hyperactivity/inattention, peer relationship problems, and prosocial behaviour. The Italian teacher-report version [60] was used in the present study. It is a brief behavioural screening questionnaire for children and adolescents aged 3–17 years. Three versions are available: teacher-report, parent-report, and child-report. Teachers were asked to answer on the basis of the child’s behaviour over the last six months of the current school year. Each item was scored by the teachers from 0 (“not true”) through 1 (“somewhat true”) to 2 (“certainly true”). Following the standard SDQ scoring procedure [58,59], and after reverse-scoring the five positively worded difficulty items, the Total Difficulties score was computed as the sum of the 20 items of the four difficulties subscales (emotional symptoms, conduct problems, hyperactivity/inattention, and peer relationship problems), excluding the prosocial behaviour subscale. Scores can range from 0 to 40, with higher scores indicating greater difficulties. For the present study, only the Total Difficulties score was used in the analyses.

#### 2.2.4. Self-Figure Drawing

The Self-Figure Drawing [1] is a projective measure that asks the participant to draw themselves on an A4 sheet of paper, in portrait orientation. No further instructions are given other than to draw themselves however they wish. The figure is hypothesised to represent the self and, on the basis of indicators previously validated cross-culturally [38,54,61], different forms of abuse (namely sexual, physical, and emotional abuse) can be assessed. The indicators are rated from 1 (not obvious at all) to 4 (highly obvious) by trained raters. In the present study, all drawings were coded independently by two raters, both psychologists and researchers (authors of the present study) trained in the use of the SFD for abuse screening. Teachers were not involved in the coding of the drawings at any stage. To limit potential bias, raters were blind to the teachers’ reports of neglect and to the SDQ scores while coding the drawings. Inter-rater reliability (Cohen’s kappa) for all the indicators used in the current study was 0.80 and above, and disagreements were resolved through discussion until consensus was reached.

Based on the scores for the indicators, an emotional abuse index score was calculated. The index score is the sum of the indicator scores. If three or more than three indicators were rated as highly obvious, the drawing was categorised as indicative of emotional abuse. This binary variable was used, keeping in line with previous literature [51,54]. As the threshold has not been independently validated in an Italian population, its application in the present study was intended as an operational classification for exploring the prevalence and correlates of EA indicators from SFD. The emotional abuse indicators are as follows: slanting figure, tiny head, omitted hands or arms, omitted legs or feet, monster-like figure, gross asymmetry of limbs, tiny figure, omitted eyes, shading of the face, and feet pressed together [54,62].

## 3. Data Analysis

Continuous variables are reported as means and standard deviations, and categorical variables as frequencies and percentages. Pearson’s correlations were used to assess associations between continuous variables. Associations between the SFD measures and sociodemographic variables (age and dichotomous variables) were examined using Spearman’s rank-order correlations. Independent-samples *t*-tests were used to compare children with and without EA indicators on teacher-reported neglect and SDQ scores. Analyses were performed using IBM SPSS Statistics, Version 29. Statistical significance was set at *p* < 0.05. Given that age was associated with the EA index, partial correlations between the EA index and each teacher-reported measure were also computed controlling for age. Furthermore, because children were nested within classrooms and all pupils in a classroom were rated by the same teacher, intraclass correlation coefficients (ICCs) were computed to quantify the proportion of variance attributable to classroom membership, and linear mixed-effects models with a random intercept for classroom (restricted maximum likelihood estimation) were estimated, with age and the EA index as predictors of teacher-reported neglect and SDQ Total Difficulties. All continuous variables were standardised before being entered into these models; therefore, the fixed effects can be interpreted as standardised coefficients (β). Analyses were organised into primary and exploratory analyses. The primary analyses tested H1 and H2, namely the Pearson correlations between the continuous EA index and teacher-reported neglect (H1) and SDQ Total Difficulties (H2); the family-wise error rate across these two tests was controlled using the Holm–Bonferroni procedure [63]. The age-adjusted partial correlations and mixed-effects models were treated as sensitivity analyses of the primary hypotheses. All other analyses—namely, the comparisons between children with and without EA indicators (categorical classification) on teacher-reported neglect and SDQ Total Difficulties, and the associations between SFD measures and sociodemographic variables—were considered exploratory. For these 14 exploratory tests, uncorrected *p*-values are reported together with *p*-values adjusted using the Benjamini–Hochberg false discovery rate (FDR) procedure [64].

## 4. Results

Based on the Self-Figure Drawing, 14.3% of children met the SFD criteria for emotional abuse (Table 1).

*Primary analyses.* As shown in Table 2, the continuous EA index was positively correlated with teacher-reported neglect (r = 0.117, *p* = 0.020, 95% CI [0.018, 0.214]) and with the SDQ Total Difficulties score (r = 0.108, *p* = 0.032, 95% CI [0.009, 0.205]). According to conventional benchmarks [65], both correlations are small in magnitude, corresponding to approximately 1.4% and 1.2% of shared variance, respectively, and the lower bounds of their 95% confidence intervals are close to zero. Both associations remained significant after Holm–Bonferroni correction for the two primary tests (adjusted *p* = 0.041 for both) [63].

*Sensitivity analyses.* Teacher-reported neglect and SDQ Total Difficulties were moderately correlated (r = 0.347, *p* < 0.001). Because age was positively correlated with the EA index (Table 3) and negatively correlated with SDQ Total Difficulties (r = −0.127, *p* = 0.012), the analyses were repeated controlling for age. Both associations remained significant and were slightly larger: the partial correlation between the EA index and teacher-reported neglect was r = 0.124 (*p* = 0.014, 95% CI [0.025, 0.221]), and that between the EA index and SDQ Total Difficulties was r = 0.133 (*p* = 0.008, 95% CI [0.035, 0.229]). ICCs indicated that 46.6% of the variance in teacher-reported neglect, 12.9% of the variance in SDQ Total Difficulties, and 11.5% of the variance in the EA index were attributable to classroom membership. In linear mixed-effects models accounting for both classroom clustering and age, the EA index remained a significant predictor of teacher-reported neglect (β = 0.108, *p* = 0.021, 95% CI [0.016, 0.199]) and of SDQ Total Difficulties (β = 0.142, *p* = 0.004, 95% CI [0.045, 0.240]). Age was not associated with teacher-reported neglect (β = −0.070, *p* = 0.478) but was negatively associated with SDQ Total Difficulties (β = −0.148, *p* = 0.044).

*Exploratory analyses.* Children classified as presenting EA indicators (n = 56) showed higher SDQ Total Difficulties scores than children without EA indicators, t(390) = 2.20, *p* = 0.028, but did not differ in teacher-reported neglect, t(390) = 0.23, *p* = 0.817 (Table 2). Among the sociodemographic variables, only age was significantly associated with the EA index (ρ = 0.183, *p* < 0.001; Table 3). After FDR correction across the 14 exploratory tests, only the association between age and the EA index remained significant (adjusted *p* = 0.004), whereas the difference in SDQ Total Difficulties according to the categorical EA classification did not (adjusted *p* = 0.199).

## 5. Discussion

The objective of this study was to conduct a preliminary test of the convergent validity of the Emotional Abuse (EA) index derived from the Self-Figure Drawing (SFD) in a community sample of Italian primary school children. Our data, consistent with Hypothesis H1, showed that the EA index was positively and significantly associated with teacher-reported neglect. Furthermore, in accordance with Hypothesis H2, it was also found to be positively and significantly associated with teacher-rated psychological difficulties. Regarding the first hypothesis, for the purposes of our study, neglect was chosen as a measure of convergence precisely because, while emotional abuse produces internal harm that is difficult to observe directly, its close conceptual and empirical association with neglect [6,8] means that neglect—being more readily observable by the teacher—can serve as an external correlate of the risk of emotional abuse. Several studies in the literature have found a strong association between emotional abuse and childhood neglect, leading some authors to combine these two forms of victimisation into the broader construct of psychological maltreatment [6,8]. Our data align with this line of research, identifying an association between the EA index measured by the SFD and teacher-reported child neglect. Overall, these findings appear to offer initial and preliminary support for the idea that the SFD’s emotional abuse index aligns, at least to some extent, with teacher-reported indicators of neglect obtained independently of the drawing coding, and thus provide early, albeit weak, evidence of the SFD’s potential relevance for identifying children at risk of emotional abuse. It should be noted, however, that both criterion measures were provided by the same informant (the teacher). Although the drawings were coded by independent raters blind to teacher reports, the teacher-based criteria themselves may be affected by teacher-level perceptual bias; therefore, these convergent associations should be interpreted with caution (see Section 6).

The association between the EA index and teacher-rated psychological difficulties is consistent with the existing literature, which recognises emotional abuse as a potential risk factor for children’s psychological adjustment [13,15]. Emotional abuse has been theorised as a factor that impairs a child’s capacity for emotional regulation and fosters negative internal working models of self and others, with consequences for interpersonal functioning [19,66]. Emotional abuse and neglect have also been linked to children’s proneness to shame [67], that is, a painful and global negative evaluation of the self, which differs from guilt, a feeling focused on a specific behaviour rather than on the self as a whole [68]. This self-focused form of harm may be particularly relevant to a drawing task that asks children to represent themselves. Moreover, emotional dysregulation and interpersonal difficulties have been found to mediate the relationship between childhood emotional abuse and subsequent depressive symptoms [18]. Emotional abuse and neglect have similarly been associated with a wide range of emotional, behavioural, and cognitive difficulties in school-aged children [13], and meta-analytic evidence links childhood emotional maltreatment to poorer mental health outcomes into adulthood [15]. Therefore, our data provide modest support for the EA index as a potential correlate of psychological distress in children, although the small size of this association (r = 0.108) indicates that the EA index accounts for only a small portion of the variability in teacher-rated difficulties. From a descriptive standpoint, based on the SFD criteria for emotional abuse, it is estimated that 14.3% of the children in the sample under examination fall into a risk category for emotional abuse. Comparing our results with previous literature is complicated by both differences in sample selection and differences in the outcome measures reported. Much of the existing SFD literature has relied on samples selected for high or confirmed risk rather than on general population samples, such as institutionalised children with documented histories of abuse [51], street children residing in group homes with a history of abuse [54], or hospitalised adolescents compared with healthy controls [46]. Furthermore, these studies—in addition to being based on samples that are numerically smaller than ours—typically report group differences at the level of individual indicators rather than the proportion of children classified as meeting the EA criteria, making a direct comparison of prevalence impossible. Given that emotional abuse is the most common form of child abuse in Italy [11,12], the 14.3% figure might be considered rather low. However, meta-analytic evidence indicates that prevalence estimates of child emotional abuse vary considerably depending on the source of information, ranging from approximately 0.3% in studies based on external informants or service data to over 36% in studies based on retrospective self-reports by adults [5]. Our figure of 14.3% falls between these two extremes: higher than what is typically reported by passive reporting systems based on external informants, but considerably lower than estimates based on retrospective self-reports. This intermediate result is consistent with what one would expect from a structured screening tool applied to a general school population. Nevertheless, this finding should be considered preliminary. Future studies should aim to replicate this estimate in samples that include children with independently confirmed histories of emotional abuse, which would allow for the establishment of population- and instrument-specific classification thresholds, rather than relying on thresholds developed in other cultural and clinical contexts. Furthermore, our study does not take into account aspects such as the severity, intensity, and duration of emotional abuse alongside the SFD indicators. Therefore, the present finding aggregates children who may differ considerably along these dimensions, and a more comprehensive understanding of what the 14.3% estimate represents will likely require this additional level of detail in future investigations.

The magnitude of these associations deserves explicit consideration. Correlations of 0.108–0.117 are small by conventional standards [65] and indicate that the EA index shares only about 1–2% of its variance with each teacher-reported criterion. Although they are statistically distinguishable from zero in a sample of this size, their confidence intervals are relatively wide and their lower bounds approach zero; they should therefore be read as evidence that the EA index is not unrelated to the teacher-reported criteria, rather than as evidence of substantial convergence. Several features of the design may have attenuated the observed correlations and help to place them in context. First, the EA index and the criteria were derived from different methods (a child-produced drawing coded by independent raters versus teacher ratings), and cross-method validity coefficients in psychological assessment are typically considerably lower than associations obtained within the same method [69]. Second, in an unselected community sample with a low base rate of maltreatment, the restricted range of both the EA index and the neglect scores is likely to reduce the size of correlations. Third, teacher reports capture only the observable, school-based manifestations of neglect and distress, which overlap only partly with the internal harm that emotional abuse is theorised to produce. Effect sizes of this order are also not uncommon in individual-differences research [70,71]. Nevertheless, these considerations help to explain the size of the effect rather than enlarge it: at the individual level, an association of this magnitude has very limited predictive value, and the EA index alone could not reliably discriminate between children with higher and lower levels of teacher-reported neglect or difficulties. For this reason, we interpret the present findings as preliminary, group-level support for the convergent validity of the EA index, and not as evidence of its accuracy for individual screening decisions.

Another nuance concerns the difference between the continuous EA index and the categorical classification of presence/absence. The continuous index was found to be significantly associated with teacher-reported neglect, whereas the categorical classification of EA presence—based on the established threshold of three or more very obvious indicators—did not reach statistical significance. Classification thresholds of this type are typically calibrated using known-groups or clinically selected samples, in which the base rate of confirmed abuse is considerably higher than in an unselected sample from the general population such as the one presented here. It is therefore to be expected—rather than considered anomalous—that a threshold developed under those conditions would behave slightly differently when applied to a community sample with a lower base rate. Rather than indicating a limitation of the SFD coding system itself, this pattern may more readily reflect a broader and well-recognised methodological principle: population-specific calibration is a normal and necessary step whenever a validated screening threshold is extended to a new context, and the same recalibration process would be expected for any threshold-based tool applied outside the population in which it was originally developed. In the meantime, the present data suggest that the continuous EA index may represent the most sensitive metric for research purposes in samples of this type, pending dedicated work on population-specific threshold calibration. It should also be noted that the association between the categorical EA classification and SDQ Total Difficulties was examined as an exploratory analysis and did not survive correction for multiple testing; it should therefore be interpreted with caution.

In exploratory analyses, age was also found to be positively associated with the EA index, and this association remained significant after correction for multiple testing. Although the literature is mixed regarding the association between a child’s age and the risk of experiencing emotional abuse, some data seem to point in this direction, suggesting that as children grow older, more cases of intrafamilial emotional abuse are reported [72]. However, other explanations are also possible and should be further examined in the future. In fact, while this may reflect a genuine increase in indicators of emotional abuse with age, an equally plausible explanation is that this association reflects age-related maturation in children’s drawing abilities, since older children in this sample might produce more detailed and complex drawings [43]. Importantly, controlling for age did not attenuate the associations between the EA index and the teacher-reported measures.

## 6. Limitations and Future Research Directions

Several limitations should be taken into account when interpreting these results. First, the study used a cross-sectional, correlational design, so it is not possible to draw causal conclusions about the relationship between SFD indicators of emotional abuse, on the one hand, and neglect and psychological adjustment, on the other. Future studies may therefore adopt a longitudinal perspective to test whether SFD indicators precede, follow, or co-occur with changes in these outcomes over time. Second, teachers were the sole informants for both the neglect scale and the SDQ. Because the EA index was coded by independent raters who were blind to teacher reports, its associations with the two teacher measures cannot be attributed to shared-method variance between the drawing and the criterion measures. However, both criteria remain vulnerable to teacher-level perceptual bias: for instance, a teacher who perceives a child as neglected may also tend to rate that child as showing greater psychological difficulties (a halo effect), and teachers may differ systematically in how severely they rate their pupils. Such bias cannot, by itself, produce an association with an independently coded drawing index, but it reduces the extent to which neglect and psychological difficulties can be considered two distinct, independent criteria, and it could contribute to the observed associations if teachers’ perceptions were partly shaped by child characteristics that are also reflected in the drawings (e.g., general developmental or graphic maturity). Moreover, because several children were rated by the same teacher, the data were clustered at the classroom level. Notably, almost half of the variance in teacher-reported neglect was attributable to classroom membership (ICC = 0.47), which is consistent with teacher-level rating tendencies. Although the associations remained significant in multilevel models accounting for this clustering, teacher and classroom effects could not be disentangled, as the pupils in each classroom were rated by a single teacher. Future studies should therefore include multiple informants (e.g., parents, school psychologists, and children’s self-reports), involve more than one rater per classroom to disentangle teacher-level from classroom-level effects, and, where possible, include independently confirmed indicators of neglect and psychological distress. Third, the sample under examination was selected from the general school population rather than from a group with a history of independently confirmed maltreatment; the study was therefore unable to verify validity against known groups, sensitivity, or specificity—all of which are necessary before any index based on this design can be considered for applied screening purposes. Furthermore, the children included in this study attend schools in northern Italy, which may limit the generalisability of the findings to the national population. Fourth, the age-related increase in the EA index could not be distinguished from the possible effects of maturation on the children’s drawing abilities, as noted earlier. More generally, our study did not account for the children’s graphic skills; therefore, this variable could be controlled for in future research. Fifth, we did not collect information on family socioeconomic status or on school-level resources. Given that structural conditions such as poverty may increase the risk of emotional abuse and neglect, whereas supportive school environments may act as counteracting factors that attenuate, or make less visible, its observable consequences [21,24], future studies should include these variables to examine how factors operating at different levels interact in shaping both SFD indicators and teacher-reported outcomes. Finally, the effect sizes observed, while statistically significant, were modest (r ≈ 0.11), corresponding to approximately 1–2% of shared variance. Given this effect size and the current sample size, the post hoc statistical power to detect these associations was approximately 59%, below the conventional 80% threshold. This does not invalidate the significant associations found, but it does suggest that comparable or smaller true associations may have gone undetected, reinforcing the preliminary status of these findings pending replication in larger samples.

### Practical Implications

Taking into account the limitations noted above, these results provide preliminary support for the potential use of the SFD emotional abuse index as a complementary, non-verbal screening tool in school settings. Although it is not intended as a tool for identifying cases of emotional abuse, this instrument could be useful in flagging potential at-risk cases that warrant more specific attention to possible situations of family distress. As drawing activities do not require verbal expression, they may be particularly useful with younger children or those who are reluctant or unable to discuss family difficulties directly. In practice, this could mean using high scores on the emotional abuse (EA) index as a starting point for a school psychologist or counsellor to observe a child more closely or to initiate a supportive conversation, rather than as grounds for an independent judgement of abuse. Given the modest effect sizes reported here, the SFD should be considered one of several components within a broader, multi-method screening protocol, rather than a substitute for teacher observation, structured questionnaires, or professional clinical judgement. More modestly, our study suggests that further research should be conducted into the potential use of the SFD as a screening tool for children who may have been exposed to adverse family experiences.

## 7. Conclusions

This study provided a preliminary test of the convergent validity of the Self-Figure Drawing emotional abuse index in a community sample of Italian schoolchildren. Consistent with the study’s hypotheses, the EA index showed modest but statistically significant associations with two independent, teacher-based indicators: reported neglect and global psychological difficulties. These findings offer initial support for the use of the SFD as a non-verbal correlate of emotional maltreatment outside the cultural contexts in which the instrument was originally developed and validated. At the same time, the modest effect sizes, the reliance on a single informant, and the exploratory status of several secondary findings indicate that these results should be interpreted as preliminary rather than conclusive. Before the SFD emotional abuse index can be considered for applied use in Italian school settings, further validation work is needed, particularly studies employing multiple informants, known-groups comparisons, and adequately powered samples. Within these limits, the present findings contribute initial cross-cultural evidence for the SFD as a candidate screening tool and provide a basis for more targeted validation research going forward.

## Figures and Tables

**Table 1:** Descriptive statistics of the study variables.

Variable	M (SD)/*n* (%)
Age	8.93 (0.84)
**Gender**	
Female	192 (49)
Male	200 (51)
Special Needs	54 (13.8)
Learning Disorders	22 (5.6)
Physical Disability	4 (1)
Cognitive Disability	3 (0.8)
Teacher’s Report on Neglect	1.45 (2.55)
**Self-Figure Drawing**	
Emotional Abuse (EA) Index	15.04 (3.02)
Presence of EA	56 (14.3)
SDQ Total	6.28 (5.94)

**Table 2:** Correlations between Self-Figure Drawing indicators and teachers’ reports of neglect and SDQ scores.

Variables	EA Index	EA
Teacher’s Report on Neglect	0.117 *	0.012
SDQ Total	0.108 *	0.111 *

* *p* < 0.05.

**Table 3:** Correlations (Spearman’s rho) between Self-Figure Drawing indicators and sociodemographic variables.

Variables	EA Index	EA
Age	0.183 **	0.048
Gender	0.022	0.05
Special Needs	0	0
Learning Disorders	−0.042	−0.068
Physical Disability	0.09	−0.041
Cognitive Disability	−0.033	−0.036

** *p* < 0.01.

## Data Availability

The data presented in this study are available on request from the corresponding authors.
